# Silicon-Nanographite Aerogel-Based Anodes for High Performance Lithium Ion Batteries

**DOI:** 10.1038/s41598-019-51087-y

**Published:** 2019-10-10

**Authors:** Manisha Phadatare, Rohan Patil, Nicklas Blomquist, Sven Forsberg, Jonas Örtegren, Magnus Hummelgård, Jagruti Meshram, Guiomar Hernández, Daniel Brandell, Klaus Leifer, Sharath Kumar Manjeshwar Sathyanath, Håkan Olin

**Affiliations:** 1 Department of Natural Sciences, Mid Sweden University, Sundsvall, SE-851 70 Sweden; 2 Centre for Interdisciplinary Research, D.Y. Patil Education Society (Deemed University), Kolhapur, 416 006 Maharashtra India; 3 Department of Chemistry - Ångström Laboratory, Uppsala University, Box 538, SE-751 21 Uppsala, Sweden; 4 Electron Microscopy and Nano-Engineering, Applied Materials Science, Department of Engineering Sciences, Uppsala University, Box 534, 75121 Uppsala, Sweden

**Keywords:** Batteries, Batteries

## Abstract

To increase the energy storage density of lithium-ion batteries, silicon anodes have been explored due to their high capacity. One of the main challenges for silicon anodes are large volume variations during the lithiation processes. Recently, several high-performance schemes have been demonstrated with increased life cycles utilizing nanomaterials such as nanoparticles, nanowires, and thin films. However, a method that allows the large-scale production of silicon anodes remains to be demonstrated. Herein, we address this question by suggesting new scalable nanomaterial-based anodes. Si nanoparticles were grown on nanographite flakes by aerogel fabrication route from Si powder and nanographite mixture using polyvinyl alcohol (PVA). This silicon-nanographite aerogel electrode has stable specific capacity even at high current rates and exhibit good cyclic stability. The specific capacity is 455 mAh g^−1^ for 200^th^ cycles with a coulombic efficiency of 97% at a current density 100 mA g^−1^.

## Introduction

Rechargeable lithium-ion batteries are popular devices for energy storage due to their high energy density, excellent environmental compatibility, long life cycles, and low self-discharge rates. Lithium-ion batteries are widely used in mobile applications, electric vehicles, and other devices^1–4^. However, there is an ever-increasing demand to develop lithium-ion batteries with lower weight, larger capacity, and longer cycle life. Traditional lithium-ion batteries use graphite as anode material, which has a maximum theoretical capacity of 372 mAh g^−1^ and poor capacity retention at a high current density^5–9^. Silicon has attracted considerable attention as one of the most promising anode materials due to its high specific capacity (~4200 mAh g^−1^ for the Li_22_Si_5_ phase and ~3579 mAh g^−1^ for the Li_15_Si_4_ phase), which is approximately 10~12 times greater than traditional graphite anodes. Silicon (Si) also has a low discharging potential (0~0.4 V vs Li/Li^+^) compared to other anode materials. Furthermore, Si is abundant, inexpensive, and environmentally friendly, making it an attractive anode material for lithium-ion batteries^10–12^.

Despite these advantages, Si-based lithium-ion batteries suffer from large volume expansion during the lithiation process, poor electrical conductivity, and short life cycles^10–13^. In the fully lithiated state of Si, i.e. the Li_22_Si_5_ phase, drastic structural changes appear (an approximate 400% volume expansion)^14,15^. This volume expansion leads to pulverization of the Si particles causing rapid degradation of the electrical connectivity of the electrode^16^. Furthermore, when Si expands and contracts, the solid-electrolyte interphase (SEI) film on the outer surface of the electrode breaks up in a cyclic manner, resulting in the continual formation of new insulating SEI film and eventually poor electrical conductivity^17^.

Over the past decade, great attention has been paid to improving the performance of Si-based anode materials by preparing amorphous structures, porous architectures, nanometer scale particle size, and sandwiched designs, among other methods^7,11–14^. One effective way to overcome the limitations of Si-based anode materials is preparing Si/carbon composites by coating carbon layers on the Si or incorporating Si into the carbon matrix^18,19^. However, most of the aforementioned methods involves complex processes and require expensive equipment, resulting in expensive synthesis and greater overall costs, thus limiting the practical application of Si in lithium-ion batteries.

In the present work, Si nanoparticles were grown on nanographite flakes by aerogel fabrication route from Si powder and nanographite using polyvinyl alcohol (PVA) by a simple, cost-efficient, and scalable method, which does not require expensive equipments for the synthesis. PVA is a synthetic water-soluble polymer with good biodegradability, and biocompatibility, and is nontoxic and environmental friendly^20^. Nanographite, in turn, is used as an additive to enhance the electrical conductivity of the aerogels by connecting Si particles onto and between the nanometer thick flakes. In this study, we show that electrodes prepared based on this structure show high specific capacity and cycling stability, thus being a potentially cost-effective method for Si-based anodes.

## Experimental Procedure

### Materials and methods

PVA (Average molecular weight: 9000–10000) and sodium alginate were purchased from Sigma Aldrich. Nanographite (NG) was produced using a large-scale tube shear process that allows the high volume and low-cost production of nanographite-based devices according to routes described before^21–23^, without further modification. NG is a mixture of graphene, multilayer graphene, and graphite nano platelets. The platelets are less than 100 nm thick. Si powder, with particle size ~1 μm, was obtained from VestaSi Europe AB.

In a typical procedure, 0.25 g of Si was dispersed in PVA solution (2 wt%) under vigorous stirring for 30 min using Ultra-Turrax T25 with an S 25 N-10 G shear head at 10 k rpm. PVA is used as dispersant for this purpose because it is a synthetic water-soluble polymer, effective in film forming, emulsifying, and has an adhesive quality with high mechanical strength and forms a gels like structure when dissolved in water^20^. Then, 0.5 g of exfoliated nanographite was added to the suspension and stirred for 30 min to form a Si/NG suspension. The suspension was stirred during heating at 90 °C using a magnetic stirrer at 1200 rpm to form Si-nanographite hydrogels. The hydrogels were washed several times with distilled water. The hydrogels were then freeze dried at −30 °C for 24 h and finally heated in a tube furnace at 800 °C for 2 h in a nitrogen atmosphere to form the Si-nanographite aerogels (SNGA). Freeze drying of the hydrogel was carried out to create micropores. Micropores are formed in the hydrogel due to the reduced entropic effect and subsequently improved heat transfer during freezing. Specifically, freeze drying is used as it is a simple and clean process without the need of any porogens and organic solvents^24^.

### Material characterization

Structural characterization was conducted using X-ray diffraction (XRD, Bruker D2 phaser) with Cu-Kα (λ = 1.54184 Å) radiation in the 2θ range 10° to 80° with the step width 0.01°. Raman spectra of the samples were obtained using a Raman microscope (Horiba XploRA PLUS, laser excitation at 532 nm) in a frequency range of 50–3000 cm^−1^. The microstructure of the SNGA and surface morphology of the electrodes was investigated using a field emission scanning electron microscope at 2 kV (FESEM; MAIA3, TESCAN) and a transmission electron microscope at 300 kV (Tecnai F30). Thermogravimetric analysis of SNGA and Silicon was performed using the instrument Mettler Toledo TGA-1 in order to calculate the weight percentage of silicon in the SNGA structure. The sample was heated to 850 °C in nitrogen atmosphere at 20 °C/min rate, followed by a 10 min isotherm at 400 °C. Afterwards, the measurement was changed to oxygen atmosphere and heated from 400 to 1100 °C at 20 °C/min heating rate.

### Electrochemical measurement

The SNGA, nanographite, and sodium alginate (as a binder) were mixed at a weight ratio of 60:30:10 using Ultra-Turrax T25 with an S 25 N-10 G shear head at 10 k rpm for 1 h. Sodium alginate was selected as a binder due to its rich content of carboxylic groups, high Young’s modulus, and electrochemical stability, which significantly enhances the columbic efficiency, specific capacity, and cycle stability^25,26^. The mixture of SNGA, NG and binder was deposited on copper foil (1 mg cm^−2^) to prepare the electrodes (label: SNGA/NG). Two reference electrodes were prepared by mixing i) nanographite and sodium alginate binder (weight ratio 90:10) labeled as NG and ii) silicon, nanographite and sodium alginate (weight ratio 21:69:10) labeled as SNG. The half cells of these electrodes were assembled in a glove box filled with highly pure argon gas (H_2_O < 0.1 ppm and O_2_ < 0.1 ppm). Lithium metal foil was used as reference and counter electrode. The electrolyte used was LP40, that is, 1 M LiPF_6_ in a mixture of ethylene carbonate (EC) and diethyl carbonate (DEC) in a 1:1 weight ratio. Celgard 2325 was used as a separator.

Cyclic voltammetry (CV) tests were performed between 0.01 and 2.0 V at a scan rate of 0.1 mV s^−1^ using a VersaSTAT 4 Potentiostat. Galvanostatic charge-discharge tests of the cells were performed using a LabVIEW-based PXI system in a voltage range between 0 and 1.5 V at various current densities. The capacities and current densities were calculated based on the weight of the active materials (total weight of SNGA + NG in case of SNGA/NG electrode, weight of NG in case of NG electrode and total weight of Si + NG in case of SNG electrode) without binder. All of the electrochemical measurements were conducted at room temperature.

## Results and Discussion

### Materials analysis

**Figure 1:**
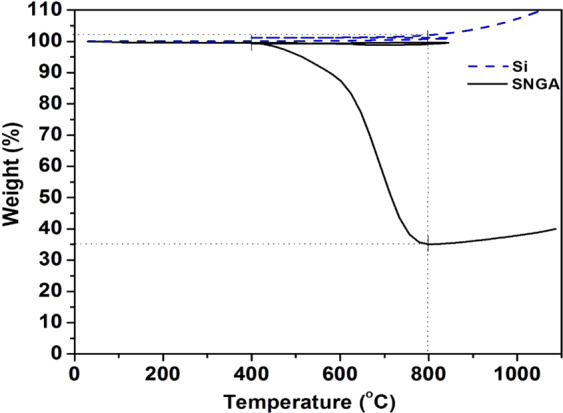
TGA curve of the silicon and SNGA.

TGA measurement of the SNGA and Silicon was performed by heating/cooling in nitrogen atmosphere 30-850-400 °C with at the rate of 20 °C/min. followed by 10 min. isotherm at 400 °C and is shown in Fig. 1. Thereafter, a switch to oxygen atmosphere was made, followed by heating from 400 to 1100 °C with the 20 °C/min. heating rate. TGA measurement of the silicon was carried out to determine the exact percentage of the silicon in the aerogel composite. TGA measurement of silicon shows small gain in weight indicating oxidation of silicon with the formation of SiO_x_. From the TGA curve of the SNGA sample, it is observed that the there is no appreciable weight loss in the nitrogen atmosphere. In the oxygen atmosphere, there is a weight loss of 64% started at 400 °C and ends at 800 °C, corresponds to combustion of graphite^27^. Based on this result, the content of silicon in the SNGA structure is calculated to be 34.65%. The SNGA, nanographite, and sodium alginate binder were mixed at a weight ratio of 60:30:10 to prepare the electrode. Hence, percentage of silicon is 20.79% in the final electrode.

**Figure 2:**
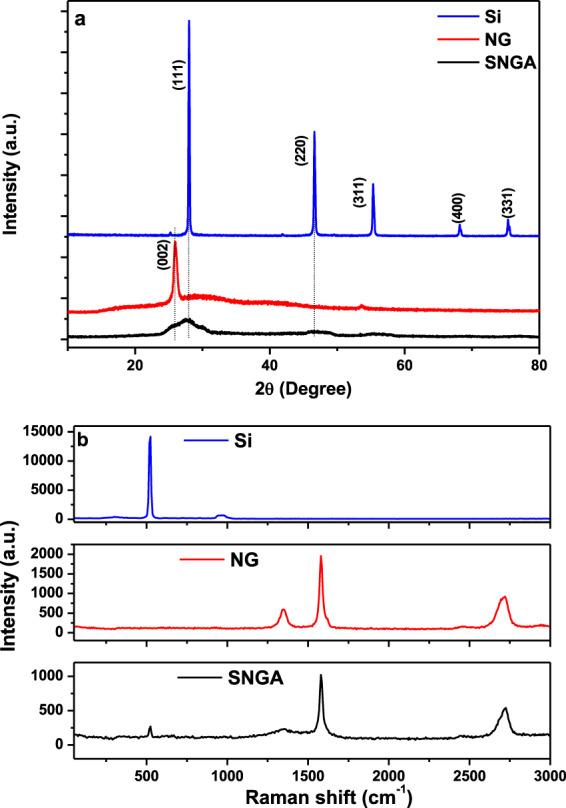
(**a**) XRD pattern and (**b**) Raman spectra of Si, NG, and SNGA.

The XRD patterns of Si, NG, and SNGA are shown in Fig. 2a. For Si, five diffraction peaks are observed at 28.3°, 47.0°, 55.8°, 68.4°, and 75.5°. These diffraction peaks are related to Bragg’s reflections from the (111), (220), (311), (400), and (331) planes of the Si phase (JCPDS no. 27-1402), respectively. For NG, a diffraction peak is observed at 26.1 that corresponds to the (002) plane of graphite^28^. The significant reduction of the characteristic peak of silicon and nanographite observed in the XRD pattern of SNGA may be due to the presence of amorphous substances covering the structure. The thickness and uniformity of amorphous substances blocks the X-rays from reaching the crystalline silicon particles and nanographite flakes suppressing their peaks in corresponding XRD pattern^29^.

Raman spectroscopy was carried out to study the structural aspects of Si, NG and SNGA samples. Figure 2b shows the Raman spectra of Si, NG, and SNGA. Both Si and SNGA shows the peak centered at 523 cm^−1^ which corresponds to crystalline Si^30^. Thus, it is clear that there is no phase change of Si during the aerogel preparation process. The peaks observed at 1346, 1579, and 2720 cm^−1^ of the samples NG and SNGA are related to graphite. These peaks correspond to the D, G, and 2D bands, respectively^31^. Similarly, there is no change in graphite phase.

**Figure 3:**
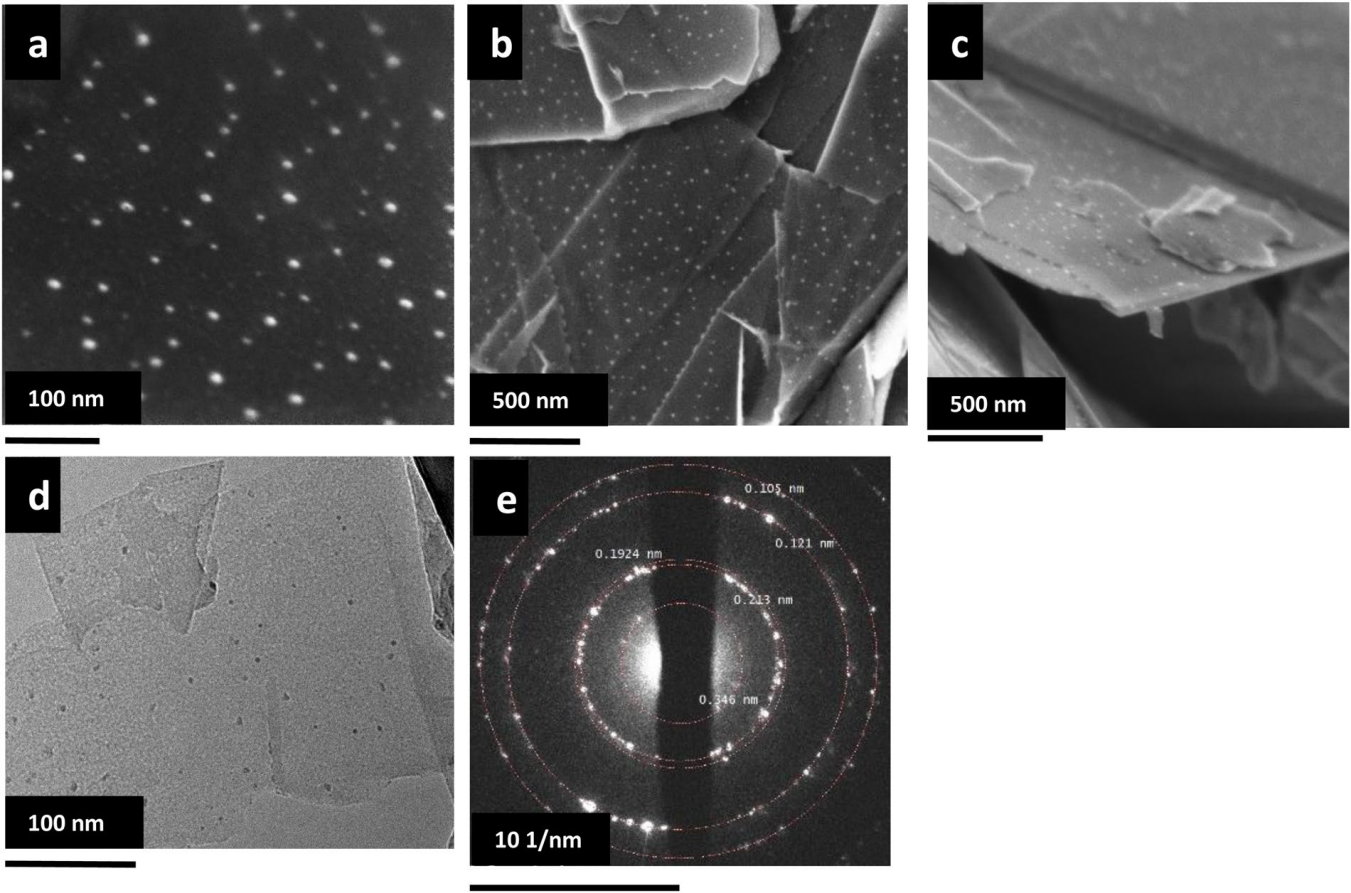
SEM images (**a**–**c**), TEM image (**d**) and corresponding SAED pattern (**e**) of SNGA.

SEM images of the SNGA structure are shown in the Fig. 3a–c revealing the presence of nanoparticles on the nanographite flakes. To further analyze these nanoparticles; TEM images of the SNGA structure were taken. Figure 3d shows the TEM image of the SNGA. TEM analysis further reveals, in SNGA structure the nanoparticles covering the nanographite flakes are of silicon/silica; Both SEM EDS and TEM EDS shows presence of silicon and oxygen, however in SEM EDS oxygen is at higher ratio indicating silicon to be of SiO_x_ form (See supplementary information). The selected area electron diffraction (SAED) pattern shown in Fig. 3e exhibits the rings made up of discrete spots. The rings corresponds to reflections with d spacing 0.346 nm, 0.213 nm, 0.1924 nm, 0.121 nm and 0.105 nm. The reflections with d spacing 0.346 nm, 0.213 nm, 0.121 nm and 0.105 nm corresponds to (002), (100), (110) and (201) planes of graphite respectively while the reflection with d spacing 0.1924 nm is due to (220) plane of silicon implying presence of nanographite and silicon. One likely mechanism describing the formation of particles can be understood by a silane route. PVA undergoes pyrolysis at about 230 °C, decomposes rapidly and further reacts with silicon in nitrogen atmosphere forming a gaseous silane (SiH_4_). The silane gas diffuses over the graphite flakes and further undergoes thermal degradation due to high temperatures forming the silicon nanoparticles over the nanographite flakes (as seen in the SEM and TEM image)^32,33^. However, after aerogel preparation process, part of the silicon nanoparticles undergo oxidation when air comes inside the tube furnace forming SiO_x._ Further, the NG aerogel was prepared by the same procedure described above. These structures does not show presence of any nanoparticles on the nanographite flakes (see supplementary information Fig. 4). From SEM image, the estimated amount of silicon nanoparticles in the SNGA structure is around 10.37(2) weight %. (see supplementary information for details). Hence, the amount of silicon nanoparticles in the SNGA/NG electrode is 6.2(2) weight %. Therefore, in the SNGA/NG electrode out of 20.79 silicon, 6.2 is nanosized silicon and remaining 14.59 is silicon microparticles.

**Figure 4:**
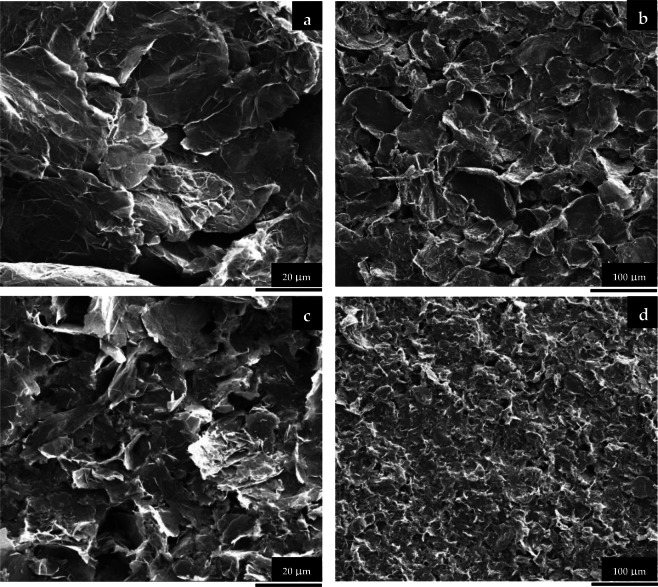
SEM images (**a**,**b**) of the NG and (**c**,**d**) SNGA/NG electrodes at different magnifications.

### Electrode analysis

SEM images of the NG and SNGA/NG electrodes at 20 μm and 100 μm are shown in Fig. 4a–d, respectively. From Fig. 4a,b, the NG electrode contains nanographite flakes of different sizes that are stacked over each other. A few pores are observed in these structures, which are formed due to the stacking of nanographite flakes of different sizes. However, Fig. 4c,d demonstrate that the SNGA/NG electrode that contains Si-nanographite aerogels with nanographite shows a large number of small and large pores with relatively smaller nanographite flakes.

**Figure 5:**
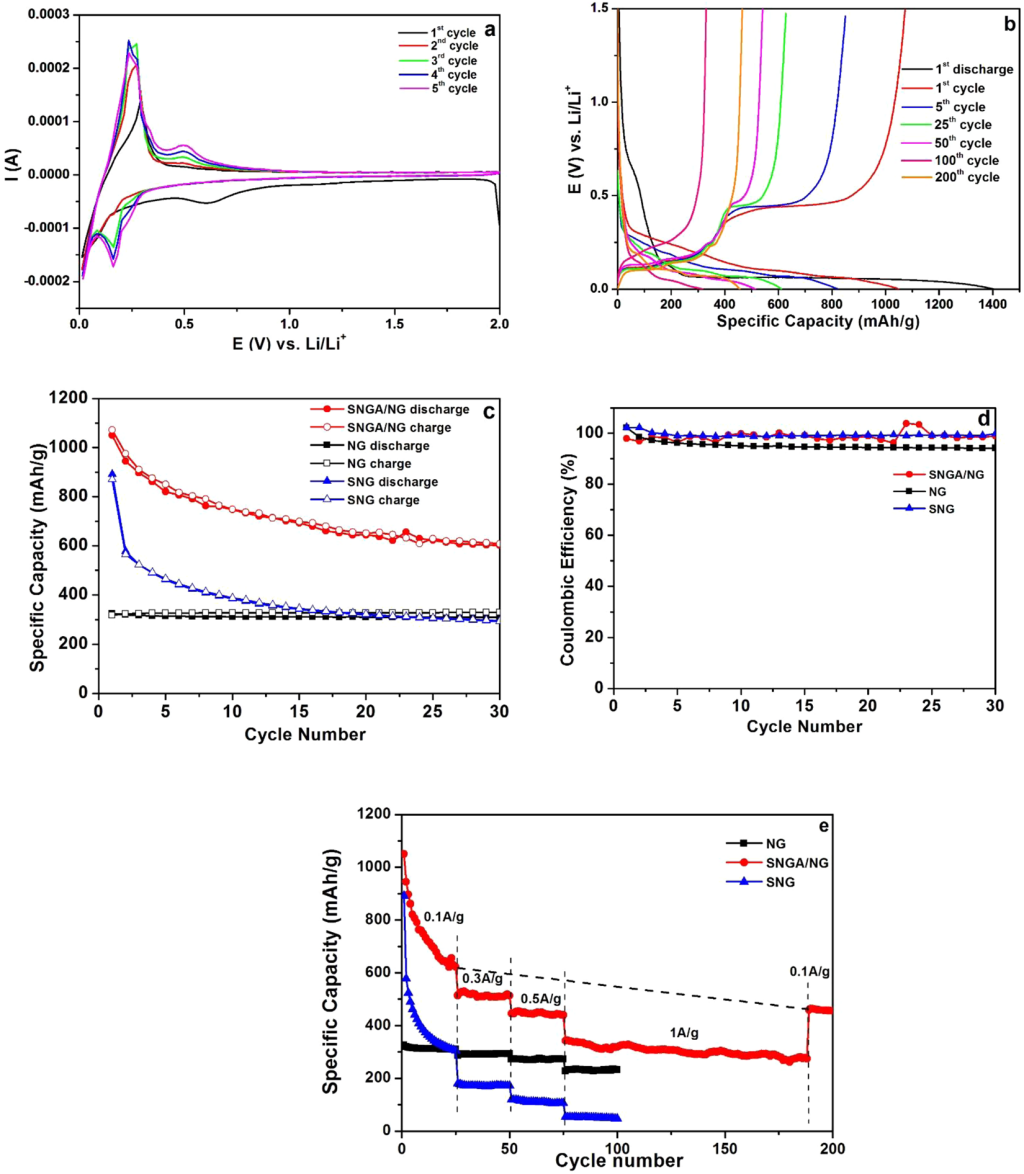
(**a**) Cyclic voltammograms of the SNGA/NG electrode at a scan rate of 0.1 mV s^−1^. (**b**) Typical charge-discharge profiles of the SNGA/NG electrode at the 1^st^ (0.09 C), 5^th^ (0.09 C), 25^th^ (0.27 C), 50^th^ (0.45 C), 100^th^ (0.9 C), and 200^th^ (0.09 C) cycles. (**c**) Specific capacities of the SNGA/NG, NG and SNG electrodes at a current density of 100 mA g^−1^ (equivalent to 0.09 C). (**d**) Coulombic efficiency vs cycle number of the SNGA/NG, NG and SNG electrodes. (**e**) Rate performance of the SNGA/NG, NG and SNG electrodes at different current densities 0.1 A g^−1^ (0.09 C), 0.3 A g^−1^ (0.18 C), 0.5 A g^−1^ (0.45 C), and 1 A g^−1^(0.9 C).

The charging/discharging mechanism of the SNGA/NG electrode (as an anode) for lithium-ion batteries was investigated using CV. The CV measurements were performed on half cells in a voltage range between 0.01 to 2.0 V at a scan rate of 0.1 mV s^−1^ for five cycles as shown in Fig. 5a. A cathodic peak is observed from 0.78 to 0.46 V in the first scan, which is attributed to the formation of a thin SEI layer. This peak disappeared in the successive cycles, indicating the formation of an SEI in the first cycle. Another cathodic peak is observed in the first cycle at 0.01 V, which is characteristic of lithiation of crystalline and amorphous Si. This splits into two peaks at 0.16 and 0.01 V in the second cycle and becomes sharper in the further cycles^34^. These peaks belong to the lithiation of Si and formation of a Li-Si alloy. In the first cycle, one anodic peak at 0.29 V is characteristic of amorphous Si and splits into two peaks at 0.29 and 0.49 V in the second cycle and becomes sharper in the further cycles. These peaks correspond to the phase transition from Li-Si alloy to Si. After the first cycle, the intensities of the cathodic and anodic peaks increases, indicating improvement in the Li insertion and extraction kinetics.

To study the electrochemical performance of the SNGA/NG electrode, galvanostatic charge-discharge measurements were conducted at a current density of 100 mA g^−1^ (equivalent to 0.09 C) in a voltage range of 0 to 1.5 V. Galvanostatic charge-discharge measurements of the NG and SNG electrodes were also conducted at the same current density and voltage range for comparison. The typical charge-discharge profiles of the SNGA/NG electrode at the 1^st^, 5^th^, 25^th^, 50^th^, 100^th^, and 200^th^ cycles is shown in Fig. 5b. The current density in the 1^st^, 5^th^, 25^th^ cycles was 0.1 A g^−1^ and that of the 50^th^, 100^th^, and 200^th^ cycles was 0.3 A g^−1^ (0.27 C), 1 A g^−1^ (0.9 C), and 0.1 A g^−1^, respectively. The first discharge shows two slopes between 0.78–0.46 V and 0.16–0.01 V, which can be correlated with the cathodic peaks observed at the same position in the discharge of the first cycle in CV. This is largely responsible for the electrode’s capacity. The slope between 0.78 and 0.46 V (corresponding to the formation of the stable SEI layer) disappears in subsequent cycles while a slope between 0.16 and 0.01 V is present in the discharge of subsequent cycles. From the charge-discharge profiles of the SNGA/NG electrode, the plateaus between 0.16–0.01 V are present up to 200 cycles, indicating that lithiation-delithiation occurs in the Si particles without pulverization and disintegration from the current collector.

Figure 5c,d shows the cycling performance and corresponding coulombic efficiency of the SNGA/NG, NG and SNG electrodes at a current density of 100 mA g^−1^. In the first cycle, the SNGA/NG electrode has a discharge capacity of 1050 mAh g^−1^ and a charge capacity of 1072.2 mAh g^−1^ with a coulombic efficiency of 97.9%, while the NG electrode has a discharge capacity of 325.8 mAh g^−1^ and a charging capacity of 318.7 mAh g^−1^ with a coulombic efficiency of 102.2% and the SNG electrode has a discharge capacity of 890.7 mAh g^−1^ and a charging capacity of 870.6 mAh g^−1^ with a coulombic efficiency of 102.3%. In the second cycle, the discharge capacity decreases to 944.4 mAh g^−1^, 319.8 mAh g^−1^ and 577.4 mAh g^−1^ for SNGA/NG, NG and SNG, respectively. Finally, in the 30^th^ cycle, the discharge capacity decreases to 603.1 mAh g^−1^, 310 mAh g^−1^ and 293.7 mAh g^−1^ for SNGA/NG, NG and SNG, respectively. The specific capacity of the SNGA/NG electrode is higher than the NG and SNG electrodes in all the cycles. The electrodes SNGA/NG and SNG has the same percentage of silicon but there is a significant difference in their capacities and capacity retention.

The charge-discharge studies of the SNGA/NG, NG and SNG electrodes were conducted at different current densities of 0.1 A g^−1^ (0.09 C), 0.3 A g^−1^ (0.27 C), 0.5 A g^−1^ (0.45 C), and 1 A g^−1^ (0.9 C) and the corresponding results are provided in Fig. 5e. The specific capacities of the SNGA/NG, NG and SNG electrodes in the 25^th^ cycle are 622.5 mAh g^−1^, 310.2 mAh g^−1^ and 304.8 mAh g^−1^ respectively, at a current density of 0.1 A g^−1^. After increasing the applied current density to 0.3 A g^−1^ (after 25 cycles), the capacity falls by 17% in the SNGA/NG electrode, 7% in the NG electrode and 41% in the SNG electrode, remaining almost constant afterwards. When the applied current density increases to 0.5 A g^−1^ (after 50 cycles), there is a decrease in the specific capacity of 13%, 7% and 30% in the SNGA/NG, NG and SNG electrodes, respectively, remaining constant thereafter. Whereas at higher applied current densities (1 A g^−1^), there is a 22%, 20% and 49% decrease in the specific capacity in the SNGA/NG, NG and SNG electrodes, respectively, which remains constant. At 100^th^ cycles, the capacity of the SNGA/NG electrode is significantly larger than that of the NG and SNG electrodes. Further, cyclic stability measurements were conducted on the SNGA/NG electrode at an applied current density of 1 A g^−1^ up to 189 cycles and continued up to 200 cycles at 0.1 A g^−1^. The corresponding results are shown in Fig. 5e. When the applied current density decreased to 0.1 A g^−1^ (after 189 cycles), 74% specific capacity is recovered and stable capacity is delivered for the SNGA/NG electrode. This indicates that Si continues to contribute to the specific capacity of the electrode up to 200 cycles.

The specific capacity of the SNGA/ NG electrode for the first cycle is 1050 mAh g^−1^ (that is almost equivalent to the theoretical capacity 1084 mAh g^−1^ calculated based on the weight of the silicon and nanographite) which decreases to 603.1 mAh g^−1^ after 30 cycles measured at the current density of 0.1 A g^−1^. The capacity retention of SNGA/NG electrode is 57% for the 30^th^ cycle. Comparing it with previous studies of pristine silicon, milled silicon and heat treated silicon that show capacity retention of 33%, 32% and 52% respectively for 30^th^ cycle measured at the current density of 0.1 A g^−1^ ^35^. Further for 100^th^ cycle, the capacity retention for SNGA/NG is 52% while for pristine silicon, milled silicon and heat treated silicon the capacity retention is 17%,31% and 19% respectively^35^.

Jiang *et al*.^29^ have synthesized Si nanoparticles (size ~10 nm) via acid-etching Al-Si alloy powder and further prepared the Si/GO paper followed by thermal reduction at 700 °C. The electrochemical study reveals, the said electrode has capacity fade of 47% from initial capacity (3200 mAh g^−1^ to 1500 mAh g^−1^ for 3^rd^ cycle). The capacity was calculated based on the weight of active silicon particles only. Based on these calculations, the SNGA/NG electrode, has capacity of 2365 mAh g^−1^ after 100 cycles which is 58% higher than this report^29^. Lyu *et al*. has prepared silicon based gel in which Si core is covered with phytic acid shell layers through a facile high-energy ball milling method. The capacity was calculated based on the weight of active silicon particles. The gel based electrode has the capacity of 1300 mAh g^−1^ at 0.42 A g^−1^ while SNGA/NG electrode has shown the capacity of 1915 mAh g^−1^ at 0.5 A g^−1^ after 50^th^ cycle. which is 47% higher than gel electrode^36^.

In comparison with the above results, SNGA/NG electrode prepared using micron sized silicon particles have shown much better specific capacity and capacity retention. SNGA is prepared by simple, scalable, and cost-efficient method as compared with the methods explained above. The specific energy of SNGA/NG electrode was found to be 787 Wh kg^–1^ for the first cycle and for 200^th^ cycle, the energy density was 341.25 Wh kg^−1^ which is significantly higher than commercial automotive batteries based on the Si anodes^37^.

From Fig. 5c, it is observed that for 30^th^ cycle, the specific capacity of SNGA/NG and NG electrode is 603.1 mAh g^−1^ and 310 mAh g^−1^ respectively. Considering the weight percentage of nanographite in the SNGA/NG electrode, it contributes 238.7 mAh g^−1^ in the total specific capacity of electrode while the expected contribution from nanosilicon in specific capacity is 364.4 mAh g^−1^ implying the weight of nanosilicon in SNGA/NG electrode to be 10.41% (indicated in Table 1). The actual weight of nanosilicon is 6.2% while the expected value of 10.41% falls within upper bound of geometric standard deviation in weight of nanosilicon i.e. 12.4% (see supplementary information for details). However, the decaying of capacity curve indicates this effect to come from the fracturing of the microparticles in the powder.

**Table 1:** Comparision of specific capacities based on expected and actual weight percentage of nanosilicon and nanographite.

No of cycles	Actual Specific capacity of the SNGA/NG electrode	Contribution from nanographite in specific capacity	Expected weight of nanosilicon	Expected contribution from nanosilicon in the specific capacity	Actual weight of nanosilicon	Contribution in the specific capacity based on the actual weight of nanosilicon
30	603.1 mAh g^−1^	238.7 mAh g^−1^	10.41%	364.4 mAh g^−1^	6.2%	217 mAh g^−1^
200	455 mAh g^−1^	238.7 mAh g^−1^	6.2%	217 mAh g^−1^	6.2%	217 mAh g^−1^

Analyzing further for 200^th^ cycle, the specific capacity of SNGA electrode is 455 mAh g^−1^, the expected contribution from nanosilicon in specific capacity is 218 mAh g^−1^, which corresponds to 6.2% of weight percent of nanosilicon in the final electrode. The theoretical value of the weight percentage of nanosilicon (6.2%) matches closely with the actual calculated value of 6.2%. This implies that the specific capacity is mainly due to silicon nanoparticles.

## Conclusions

A simple, scalable, and cost-efficient method for the fabrication of silicon nanoparticles attached to the nanographite flakes was studied and reported. The presence of silicon/silica nanoparticles attached to the nanographite flakes was confirmed from the SEM and TEM studies. The electrochemical measurements proves stable specific capacity even at high current rates and good cyclic stability for aerogel-based electrodes. The electrodes show a specific capacity of 455 mAh g^−1^ for 200^th^ cycles with a coulombic efficiency of 97% at a current density of 100 mA g^−1^. This performance is fully explained by the contribution of nanosilicon in the electrode. This electrode is made from a scalable and low-cost aerogel method. Electrode performance for 200^th^ cycle i.e. 455 mAh g^−1^ corresponds to 341 Wh kg^−1^ which is higher value than currently reported best value of 260 Wh kg^−1^ in literature^37^. Measured nanosilicon weight was 6.2% and matches closely to the expected weight calculated from electrochemical performance of 6.2%.

## Supplementary information

Silicon-Nanographite Aerogel-Based Anodes for High Performance Lithium Ion Batteries Supplementary Information
