# Supplementary material for: Silicon-Nanographite Aerogel-Based Anodes for High Performance Lithium Ion Batteries
Source: Sci Rep. 2019 Oct 10;9:14621. doi: 10.1038/s41598-019-51087-y (PMC6787263; doi:10.1038/s41598-019-51087-y)
Supplement: Supplementary file 1 — Silicon-Nanographite Aerogel-Based Anodes for High Performance Lithium Ion Batteries Supplementary Information [file 41598_2019_51087_MOESM1_ESM.docx]

**Silicon-Nanographite Aerogel-Based Anodes for High Performance Lithium Ion Batteries**

**Supplementary Information**

Manisha Phadatare,^1,2*^ Rohan Patil,^1*^ Nicklas Blomquist,^1^ Sven Forsberg,^1^ Jonas Örtegren,^1^ Magnus Hummelgård,^1^ Jagruti Meshram,^2^ Guiomar Hernández,^3^ Daniel Brandell,^3^ Klaus Leifer,^4^ Sharath Kumar Manjeshwar Sathyanath^4^ and Håkan Olin^1^

^1^Department of Natural Sciences, Mid Sweden University, Sundsvall, SE-851 70, Sweden

^2^Centre for Interdisciplinary Research, D.Y. Patil Education Society (Deemed University), Kolhapur 416 006, Maharashtra, India

^3^Department of Chemistry - Ångström Laboratory, Uppsala University, Box 538, SE-751 21 Uppsala, Sweden

^4^Electron Microscopy and Nano-Engineering, Applied Materials Science, Department of Engineering Sciences, Uppsala University, Box 534, 75121 Uppsala, Sweden

Correspondence and requests for materials should be addressed to M.P. (email: manisha.phadatare@miun.se, phy.manisha@gmail.com) or R.P. (email: rohan.anand.patil@gmail.com)

The EDS analysis of the SNGA was during the TEM measurement and the corresponding spectrum is shown in Figure S1. The spectrum shows Si K peak from the nanoparticles. Strong oxygen peak is observed.

Stray Fe and Co peaks are from pole piece and Cu peak is from the grid.


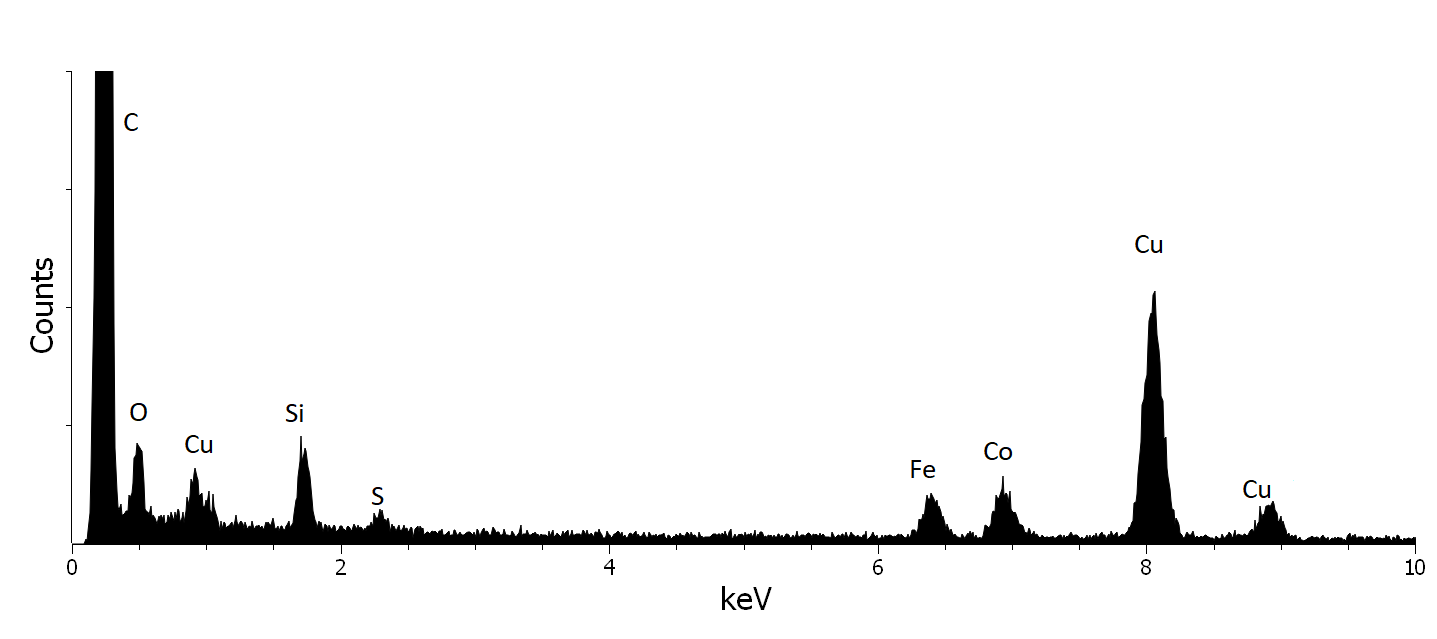


**Figure S1: EDS spectrum of SNGA taken during TEM measurement.**

For the detailed analysis of the oxygen peak, the nanographite aerogel was prepared by the same method as that of SNGA and EDS measurement of the SNGA structure and the nanographite aerogel was carried out during SEM measurement. In case of SNGA, the EDS measurement was carried on the flake containing the silicon nanoparticles and a flake in case of nanographite aerogel as shown in Figure S2a and b respectively.

Carbon peak in both the cases is normalized and the corresponding spectra is shown in Figure S3. Zoom out of oxygen peak is shown in inset of Figure S3. From the inset of Figure S3, it is observed that the oxygen count in case of SNGA structure is significantly higher than that of nanographite aerogel indicating the presence of silicon/SiO_x_ nanoparticles.


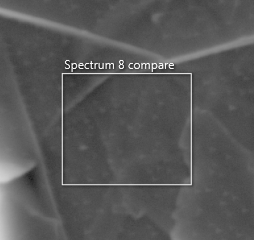

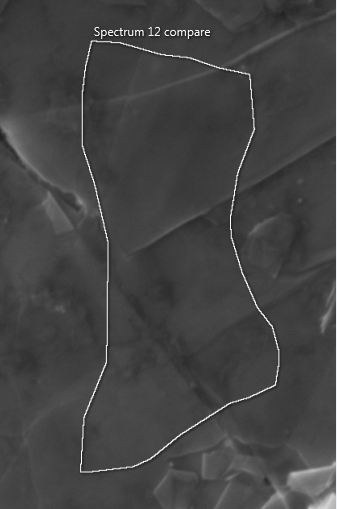


**a**

**b**

**Figure S2: Measured Area of EDS spectrum of a) SNGA and b) nanographite aerogel duing SEM measurement.**


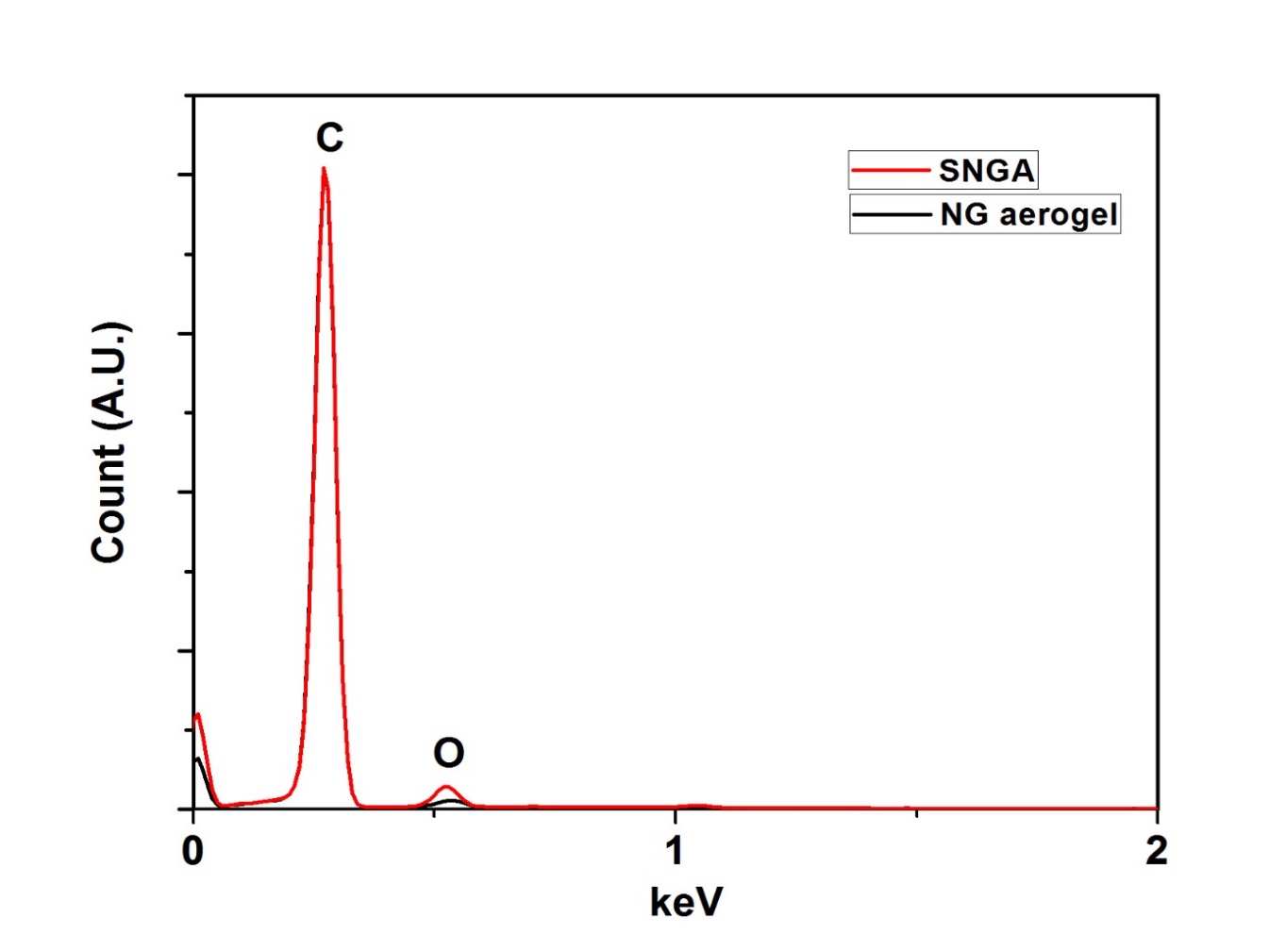

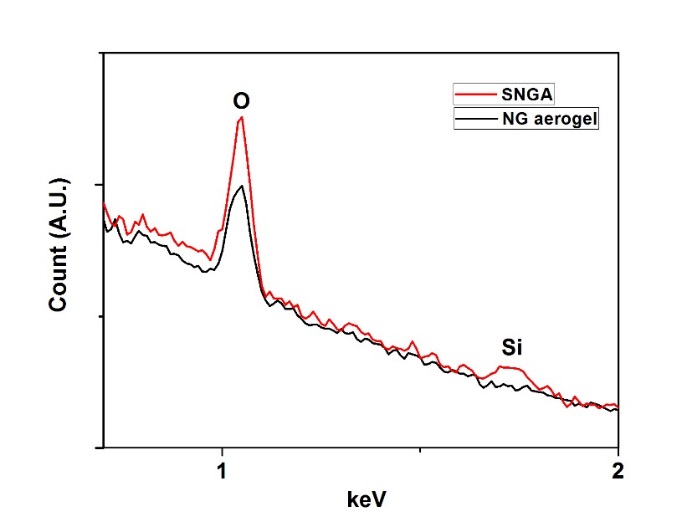


**Figure S3: EDS spectrum of SNGA taken during SEM measurement.**

The TEM images of nanographite aerogel structures were taken for comparision (Figure S4). From the TEM images, it is observed that that these structures does not show presence of any nanoparticles on the nanographite flakes as that of SNGA structure.


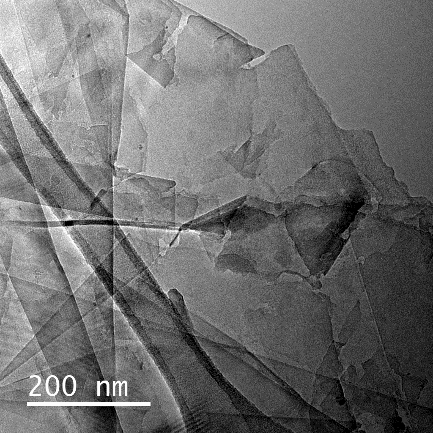

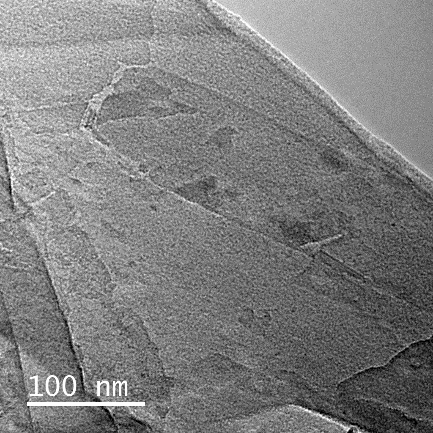


**a**

**b**

**Figure S4: TEM images of the nanographite aerogel structure at different magnifications.**


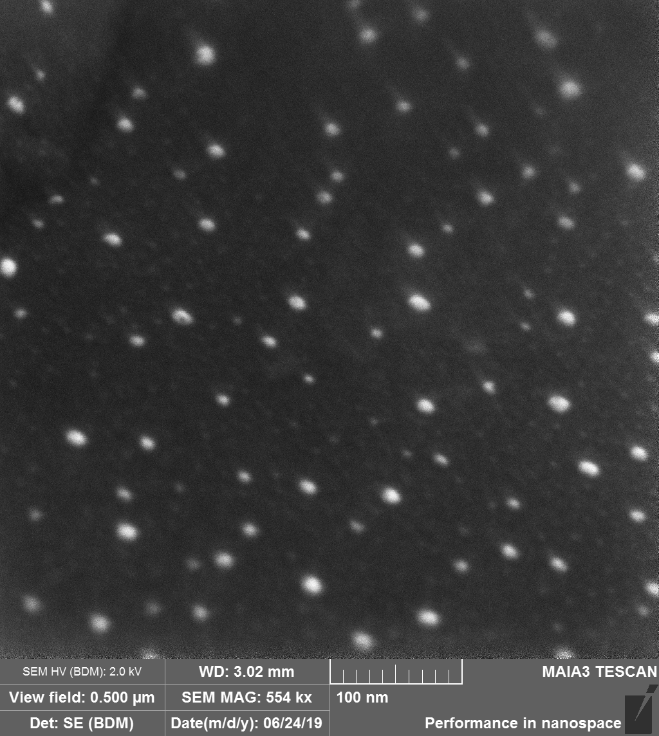


**Figure S5: SEM image of the SNGA.**

The estimated amount of the silicon nanoparticles was calculated based on the volume occupied by the nanoparticles on the nanographite flake (Figure S5). Silicon nanoparticles are assumed to be spherical to calculate the volume of the nanoparticles. Average flake area was calculated from the SEM image and based on the area, the thickness of the flake i.e. 5nm was determined from our earlier article (Sci. Rep. 9 (2019) 8966), and hence the volume. It is assumed that the silicon nanoparticles are present on both side of flake. Further, the ratio of the volume of silicon nanoparticles to that of nanographite was calculated which is further multiplied by the ratio of densities of the silicon and nanographite to determine the ratio of amount of silicon to that of nanographite. The geometric standard deviation in the volume of silicon nanoparticles was calculated and is found to be 2 (indicated in bracket in the article) and hence, the corresponding geometric standard deviation in weight percentage. Therefore, the lower bound of geometric standard deviation in weight percentage of nanosilicon will be 6.2 / 2 = 3.1 and upper bound of geometric standard deviation in weight percentage of nanosilicon will be 6.2 * 2 = 12.4.

Elemental mapping of the electrodes was done by FESEM-Energy Dispersive X-ray Spectroscopy (EDS) at 5kV. SEM images and the corresponding elemental mapping of the NG and SNGA/NG electrodes by SEM-EDS is shown in Figure S6a, b, c, d respectively. Elemental mapping shows the presence of the carbon in the NG electrode and presence of the silicon (green color) and carbon (red color) in the SNGA-NG electrode. However, this technique cannot resolve the small nanoparticles, only the big ones due to the limitations of the technique.


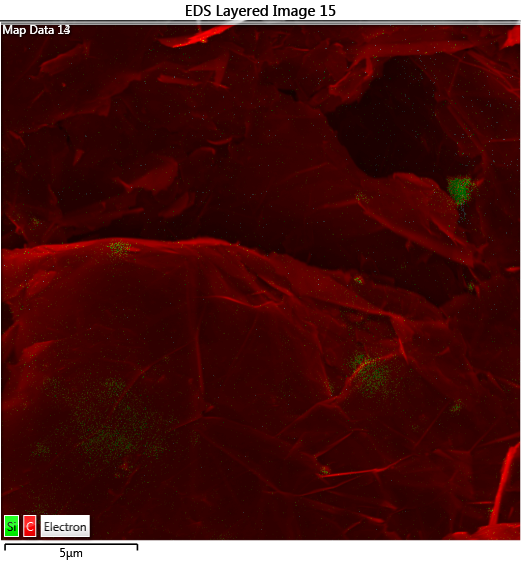

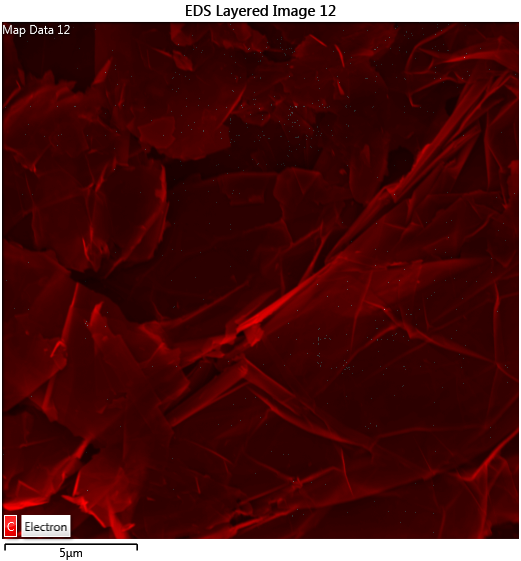

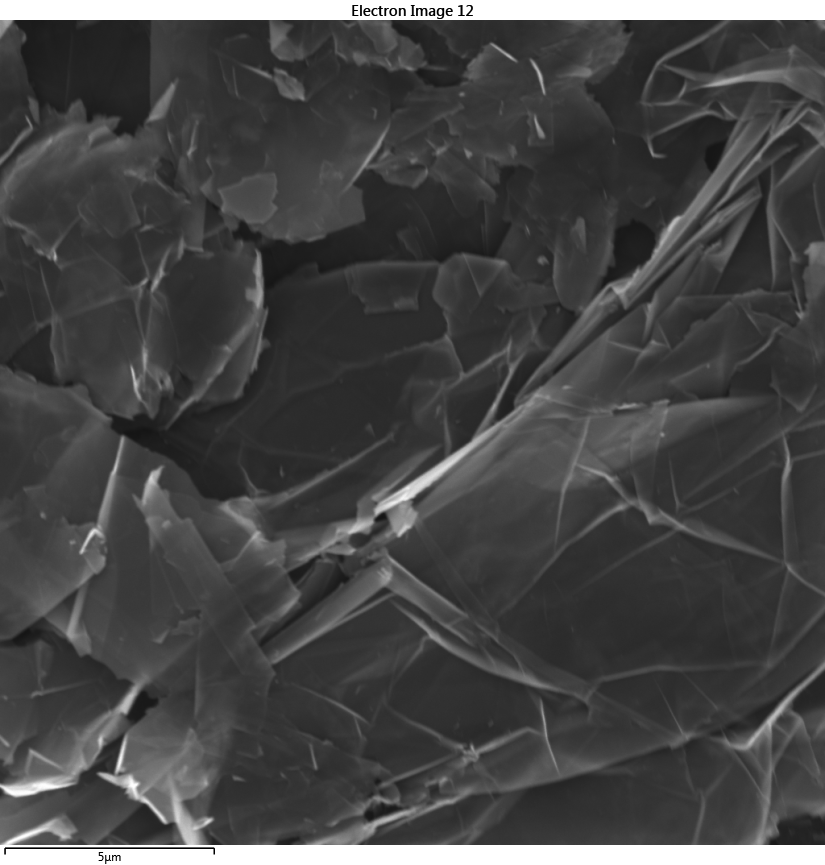

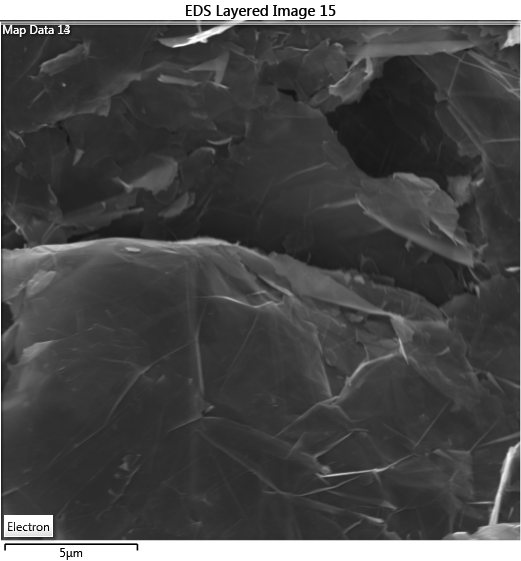


5 μm

5 μm

5 μm

5 μm

**a**

**d**

**c**

**b**

**Figure S6: SEM images (a and c) and the corresponding SEM-EDS elemental mapping (b and d) of the NG and SNGA/NG electrodes respectively.**
